# Medical aspects of terrorist bombings – a focus on DCS and DCR

**DOI:** 10.1186/2054-9369-1-13

**Published:** 2014-06-11

**Authors:** Ventsislav M Mutafchiyski, Georgi I Popivanov, Kirien C Kjossev

**Affiliations:** Endocrine Surgery and Coloproctology, Military Medical Academy, Sofia, Bulgaria; Clinic of Abdominal Surgery, Military Medical Detachment of Emergency Response, Military Medical Academy, 3 “Georgi Sofiiski” Str., Sofia, Bulgaria; Clinic of Abdominal Surgery, Military Medical Academy, Sofia, Bulgaria

**Keywords:** Terrorist bombings, Traumatic coagulopathy, Damage control resuscitation

## Abstract

Although terrorist bombings have tormented the world for a long time, currently they have reached unprecedented levels and become a continuous threat without borders, race or age. Almost all of them are caused by improvised explosive devices. The unpredictability of the terrorist bombings, leading to simultaneous generation of a large number of casualties and severe “multidimensional” blast trauma require a constant vigilance and preparedness of every hospital worldwide. Approximately 1-2.6% of all trauma patients and 7% of the combat casualties require a massive blood transfusion. Coagulopathy is presented in 65% of them with mortality exceeding 50%. Damage control resuscitation is a novel approach, developed in the military practice for treatment of this subgroup of trauma patients. The comparison with the conventional approach revealed mortality reduction with 40-74%, lower frequency of abdominal compartment syndrome (8% vs. 16%), sepsis (9% vs. 20%), multiorgan failure (16% vs. 37%) and a significant reduction of resuscitation volumes, both crystalloids and blood products. DCS and DCR are promising new approaches, contributing for the mortality reduction among the most severely wounded patients. Despite the lack of consensus about the optimal ratio of the blood products and the possible influence of the survival bias, we think that DCR carries survival benefit and recommend it in trauma patients with exsanguinating bleeding.

## Introduction

Although terrorist bombings have tormented the world for a long time, currently they have reached unprecedented levels and become a continuous threat without borders, race or age. Between 1973 and 1983, 5 075 bombing attacks with 3 689 deaths and 7 991 injuries were recorded worldwide [1]. In USA, only for 1990, there were 1 582 bombings with 222 injuries and 27 deaths, whereas for the period 1990–95 there were 15 700 incidents with 3 176 injuries and 355 deaths [2]. The peak is the notorious collapse of the World Trade Center (2001) caused 2 819 deaths, the train bombings in Madrid (2004) led to 191 deaths and 2000 injured and the attacks in London (2005) with 56 deaths and over 700 injured. In Israel, for the period 2000–2002 there were 1 116 terror-related events [3].

### Characteristic of the modern blast trauma

Approximately 64% of the combat injuries and most of the civilian ones are caused by improvised explosive devices (IEDs) [4]. In contrast to the military setting, the civilian bombings generate a higher dead/wounded ratio, 5–22 vs. 1:2–1:5 [5]. The blast trauma has unique, “multidimensional” injury pattern due to the simultaneous action of four injury mechanisms [6].

Primary blast injuries (55%) are result from the blast wave and affect the air-containing organs (lung, tympanic membrane, bowels). Usually, blast lung is manifested 24–48 after the blast and was found in 47% of the cases with immediate death, accounted for 11% mortality among the survivors [7]. Blast bowel injuries occur in only 1.2%.

Secondary blast injuries (60-70%) are caused by the secondary fragments (SFs) produced by the blast. SFs injuries are multiple and require a special awareness during the initial evaluation for several reasons [8, 9]. The multiple superficial wound can cause exsanguinating bleeding along with the possibility for multiple body cavities penetration, including vessels. The multiple injuries of the viscera require meticulous exploration of the entire abdominal cavity during the laparotomy. Additionally, there is a risk for transmission of infections such as AIDS and hepatitis [10].

Tertiary blast injuries (27-35%) are result from the body displacement by the blast wind and the structural collapse of the buildings. They lead to different kinds of blunt trauma – acceleration-deceleration injuries (usually of solid organs), fractures, traumatic amputations and head trauma. Severe brain injuries account for 71% of the immediate and 52% of the late fatalities [Fryc88].

Quaternary blast injuries constitute about 10% of all injuries and include burns, commonly associated with inhalation injuries and chemical poisoning [11].

In a comprehensive analysis comparing 906 blast victims and 55 033 patients with conventional traumas, Kluger et al., found that blast is associated with severer trauma (ISS > 16 in 28.7% vs. 10%), with multiple body regions involvement (>3, 28.3% vs. 6.2%), and higher proportion of those requiring different kind of surgery (50.8% vs. 36.6%) [12]. Blast victims (BVs) suffered more frequently from internal injuries (32% vs. 23%), open wounds (59% vs. 17%), burns (17% vs. 5%), nerves and blood vessels injuries (8% vs. 1% and 4% vs. 1%, respectively), and higher requirements for blood and blood products (5.4% vs. 1.4%) [13]. BVs required more frequent intubation (12.2% vs. 1.9%), chest decompression (5.9% vs. 1.3%), ED thoracotomy (1.2% vs. 0.2%) and angiographies (2.9% vs. 0.4%) at the admission. Blast victims were more frequently in ICU (26% vs. 7%), with higher mortality rates (6.1% vs. 2%) and rehabilitation needs (12.9% vs. 8.2%) [12].

Approximately 1–2.6% of all trauma patients and 7% of the combat casualties require a massive blood transfusion (MT) [14, 15]. Coagulopathy is presented in 65% of them with mortality exceeding 50% [14]. The risk factors for MT are systolic blood pressure < 110 mmHg, pulse > 105, hematocrit < 32, pH 7.25. In the presence of 3 or 4 of them, the risk is 70% and 85%, respectively [14]. According to Moore, the combination of ISS > 25, pH < 7.10, mean arterial pressure < 70 mmHg and T < 34° leads to coagulopathy in 98% of the cases [16].

Analysis of 82 cases, died in Iraq in Special Forces Operations, revealed that 12 of them (15%) were potentially survivable. 8/12 (66.7%) or 9.8% of all (8/82) were due to truncal hemorrhage with potential benefit from damage control resuscitation (DCR) [17].

Damage control surgery (DCS) and damage control resuscitation (DCR) were developed in the last 3 decades with aim to diminish mortality within subgroup of the patients with exsanguinating bleeding, thought as unsalvageable with the conventional approach. They are implicitly interrelated and in fact DCS can be considered as a part of DCR.

### Damage control surgery (DCS)

In 1983, Stone and al., first reported a series of 17 patients treated by a rapid control of the bleeding, open abdomen and definitive operation after physiologic stabilization. Twelve of them survived (70.9%) versus 1/12 (8.3%) treated conventionally [18]. The term “damage control” was introduced in 1993 by Rotondo and al. [19]. Subsequent studies reported 33%-89% reduction of the mortality rates [20–22]. A cumulative analysis of 1 001 damage control laparotomies revealed overall mortality 50% and morbidity rate of 40% [23].

The aim of DCS is to stop the so-called lethal triad – hypothermia (Т < 35°), acidosis (pH < 7.2, lactate level >2.5 mmol/l) and coagulopathy (INR > 1.6, аPTT > 60 sec.) [16]. The indications for DCS are exsanguinating bleeding with early onset coagulopathy, inaccessible great venous injuries (retrohepatic vena cava, pelvic veins), presence of extra-abdominal life-threatening injuries, inability for definitive repair due to mass casualty influx, lack of qualification or equipment, a need for time-consuming procedures (>90 min.) on the background of persisting hemodynamic instability [24–26]. It consists of four steps.

Ground 0 includes early recognition of the need for DCS, aggressive warming and resuscitation with blood products with subsequent rapid transfer to the operating theater [27]. The second step is a rapid control of hemorrhage and contamination (“resuscitative surgery”). Control of the contamination is best performed through a bowel stapler resection, simple suture/ligation or only clamping of the injured bowel segment. Hemorrhage control includes splenectomy, nephrectomy, temporary shunts or abdominal package. This stage should be limited to 1 hour and as a rule the abdomen is left opened. V.A.C. Abdominal Dressing System is an excellent method for temporary abdominal coverage. It provides good splinting of the abdominal wall, prevention of adhesions and lateral retraction of the fascia, which reflects in higher rate of primary facial closure, diminished risk for ACS and enteroathmospheric fistulas [28–30]. The next steps are damage control resuscitation, followed by definitive repair of the injuries after restoration of the normal physiology.

### Damage control resuscitation (DCR)

The aim of DCR is minimizing of the blood loss, improvement of the tissue oxygenation, normalization of physiology and preparation for the next stage of definitive repair of the injuries [31, 32]. The importance of this step is well-illustrated by the thought of the great surgeon lord Moynihan: “The modern operation is safe for the patient. The modern surgeon must make the patient safe for the modern operation.”

Traumatic coagulopathy (TC) occurs early after the trauma and is presented at the patient admission [33, 34]. On one hand, TC is due to shock-induced tissue damage, hypoxia and ischemia, which lead to release of pro-inflammatory cytokines, cateholamines, an activation of complement and coagulation cascade with subsequent consumption coagulopathy and hyperfibrinolysis [33, 34]. On other hand, hypothermia, acidosis and hemodilution in the cases with severe trauma are significant contributing factors. The combination of ISS > 25, pH < 7.10, MAP < 70 mmHg and Т < 34° leads to TC in 98% of the cases [16, 35]. Characteristic features of TC are hyperfibrinolysis, activation of tissue-type plasminogen activator (tPA), lack of microtrombi, thrombomodulin-protein C activation and decreased levels of protein C and antithrombin III [34, 36–38].

The failure of the standard resuscitation is attributed to massive crystalloid administration, which leads to hemodilution and reperfusion injuries, worsening the tissue perfusion, acidosis, coagulopathy and systematic inflammatory response. These events reflect in a higher frequency of ACS and multiple organ failure (MOF) [39–41].

DCR includes three major components: (1) permissive hypotension – keeping a blood pressure at 90 mmHg or palpable pulse in a conscious patient until definitive control of the bleeding [14, 41–44], (2) minimal use of crystalloids, and (3) a rapid administration of blood products (PRBC, FFP, platelets) in ratio 1:1:1 in the military practice (translated in 6 units PRBC, 6 units FFP and 1 unit apheresis platelets) [36, 45–50]. TEG/ROTEM is recommended as a rapid test for investigation of the coagulation status [51].

The administration of recombinant FVII is controversial. Although it is recommended by JTTS and European consensus, in a randomized control trial, Hauser et al. found reduced blood products use, but no survival benefit [51, 52].

Fibrinogen concentrate or cryoprecipitate are associated with improved survival should be administrated when plasma fibrinogen level is less than 1 g/l at an initial dose of 3-4 g or 50 mg/kg, respectively or in cryoprecipitate:PRBC ratio 1:1 [51, 53].

Tranexamic acid (TEA) is a competitive inhibitor of plasmin and plasminogen and is considered in the patients with trauma coagulopathy due to the recognition of the early activation of the hyperfibrinolysis [33, 34]. A retrospective military study (MATTERs) showed significantly lower mortality within the TEA group than no-TEA group (17.4% vs. 23.9%), which was more pronounced in the group of the patients with MT (14.4% vs. 28.1%, OR 7.22) [54]. The randomized placebo-controlled trial Crash-2, including 20 211 trauma patients, found significant reduced mortality in patients with massive bleeding (OR 0.85) after administration of TEA. However, this effect was evident when TEA was given within the first 1 hour (RR 0.68) and 1–3 hours (RR 0.79) after the trauma event. Importantly, the treatment given after 3 h was associated with increased risk for bleeding and death (RR 1.44, p < 0.05 [55]. On other hand, this beneficial effect may be attributed to the large sample size. Additionally, no significant differences in transfusion requirements between the two groups were recorded. TEA should be given as early as possible after trauma in all patients requiring MT. TEG/ROTEM is recommended for monitoring of the fibrinolysis and to direct the treatment [35].

There is no consensus about the optimal ratio FFP:RPBC. Most authors demonstrated that ratios greater than 1:2 were associated with reduced mortality [35, 43, 46, 47, 50]. In univariate analysis, Duchesne et al., found significantly reduced mortality rate (1:1 vs. 1:4, 26% vs. 87.5%) among the patients received >10 units of PRBC, whereas the multivariate analysis revealed relative risk for mortality of 18.9 in ratio 1:4 [47]. In a more recent study, the same author found improved 30-day survival in a greater ratio (73.6% vs. 54.8%, 1:1.2 vs. 1:4.2) [56].

The reported decrease of the mortality rates varies between 19% vs. 65% (1 vs. 1:1.4) [57], 20% vs. 31% (1:1.3 vs. 1:1.6) [50], 40% vs. 60% (>1:2 vs. <1:2) [58]. Cotton et al., revealed a striking reduction of mortality rate with 74% [59] and also a lower frequency of ACS (8% vs. 16%), sepsis (9% vs. 20%) and MOFS (16% vs. 37%) in comparison to the conventional approach [42]. In other study, the same author reported a significant reduction of resuscitation volumes, both crystalloids and blood products, and improved 24-hour and 30-day survival of the patients received DCR [60].

Despite the optimistic results, several problems should be additionally discussed. First of all, the frequency of ARDS and multiorgan failure (MOF) after MT and their effect on mortality still need to be elucidated [61]. RBC transfusion itself is associated with increased risk for ARDS and MOF [15, 62]. Meta-analysis revealed that the high plasma:PRBC ratios in a setting of MT were associated with increased ARDS risk (OR, 2.92). Nevertheless, they were associated with mortality reduction (OR, 0.38) [63]. Park et al., also found a higher frequency of ARDS in MT (8.45 vs. 5.3%), but without significant difference in the mortality (17.4% vs.16.3%) among the combat casualties from Iraq and Afghanistan [64].

Intriguingly, a recent, multicenter, prospective trial in 106 patients, based on TEG monitoring and serum lactate measurement, showed that DCR is “neither hemostatic, nor resuscitative” [65].

It should be mentioned that the reported lower mortality rates in higher ratios may be a result of so-called survival bias (SB). Due to the common practice RPBC to be administered firstly and as most deaths occurred in the first hours after the trauma, it has been suggested that the patient who live longer are likely to receive more blood product and to achieve higher FFP:PRBC ratio [66]. However, Ho et al., showed that from 21 studies, showing a survival benefit, 11 were SB-prone and 10 SB-unlikely. Five SB-unlikely studies showed no benefit in high ratios [67]. Two recent studies addressed SB and found that the improved survival is not influenced by SB. A multicenter prospective study found that high FFP:PRBC and platelets:PRBC ratios were associated with improved survival as early as 6 hours and throughout the first 24 hours [68]. Similarly, the Prospective, Observational, Multicenter, Major Trauma Transfusion (PROMMTT) study, including 905 patients from 10 Trauma centers, found decreased 6-hour mortality in high FFP:PRBC (HR, 0.31) [69].

At last, but not least, the definition of MT protocol varies between different Institutions and usually includes over 10 U PRBC/24 hours [35, 45]. Kashuk et al., proposed this definition to be changed to 10 U per 6 hours [70]. To avoid the possible selection bias, Savage et al., propose a new definition, which includes >3 U PRBC for 1 hour within the first 24 hours after the trauma [71]. The authors found that this critical administration threshold (CAT) had a stronger association with mortality vs MP. (RR, 3.58 vs. 1.82). CAT identified 75% of the deaths, while MT identified only 33%.

The need for randomized controlled trials and multicenter collaboration due to the relatively small number of patients requiring DCR in a civilian setting is obvious [14]. We are waiting with interest the results from the recent randomized trial PROPPR, which includes 608 patients and compares the effectiveness of 2:1:1 and 1:1:1 ratios [72].

## Conclusion

The unpredictability of the terrorist bombings, leading to simultaneous generation of a large number of casualties and severe “multidimensional” blast trauma require a constant vigilance and preparedness of every hospital worldwide.

DCS and DCR are promising new approaches, contributing for the mortality reduction among the most severely wounded patients. Despite the lack of consensus about the optimal ratio of the blood products and the possible influence of the survival bias, we think that DCR carries survival benefit and recommend it in trauma patients with exsanguinating bleeding.
